# Analysis of the Structural Characteristics and Psychometric Properties of the Multiple Sclerosis Intimacy and Sexuality Questionnaire (MSISQ-15): A Systematic Review and Meta-Analysis

**DOI:** 10.3390/diagnostics15222836

**Published:** 2025-11-09

**Authors:** Marta María Córdoba-Peláez, Guadalupe Molina-Torres, Anna Rutkowska, Sebastian Rutkowski, Jacobo Á. Rubio-Arias

**Affiliations:** 1 Department of Nursing, Physiotherapy and Medicine, Faculty of Health Sciences, University of Almería, 04120 Almería, Spain; mcp591@ual.es; 2 Department of Physical Education and Physiotherapy, Opole University of Technology, 45-758 Opole, Poland; a.rutkowska@po.edu.pl (A.R.); s.rutkowski@po.opole.pl (S.R.); 3 Department of Education, Health Research Center, University of Almeria, 04120 Almería, Spain; jararias@ual.es

**Keywords:** pelvic floor dysfunctions, questionnaire, validation, psychometric properties, cross-cultural adaptation

## Abstract

**Background:** The Multiple Sclerosis Intimacy and Sexuality Questionnaire (MSISQ-15) is a questionnaire designed to assess Sexual Dysfunction symptoms in multiple sclerosis patients; its first version was created in English and has been validated in 7 other languages. **Objectives:** The aim of the present study was to analyze the structural characteristics and psychometric properties of the different versions of the MSISQ-15. **Methods:** An analysis of the different versions of this questionnaire was carried out. The quality of the evidence was rated according to the Grades of Recommendation, Assessment, Development, and Evaluation (GRADE) approach. A systematic review was carried out in different databases, such as PubMed, SCOPUS, Web of Science, Dialnet, ScienceDirect, and CINAHL. The following selection criteria were considered: studies that included cross-cultural validation of the MSISQ-15, studies with a sample of the population with multiple sclerosis and suffering from sexual dysfunction, studies that presented psychometric properties with total and/or domain scores of the MSISQ-15, and studies with a sample of the population over 18 years of age. Studies that used the MSISQ-15 as an outcome measure or to validate another instrument, and studies with inconclusive results, were excluded. **Results:** A total of seven studies were analyzed with regard to structural characteristics and psychometric properties, such as reliability, internal consistency, construct validity, and criterion validity. **Conclusions:** The different versions of the MSISQ-15 are valid for use in the Polish, German, Italian, French, Turkish, Greek, and Spanish populations, and it will be necessary to adapt the questionnaire to other languages for use with patients with multiple sclerosis in other countries.

## 1. Introduction

Normal sexual function depends on several factors, including neurological factors. Therefore, sexual dysfunction (SD) is one of the most common manifestations of disorders affecting the central or peripheral nervous system, including multiple sclerosis (MS) [1,2], a chronic, autoimmune, and neurodegenerative disease that affects the central nervous system, deteriorating the white matter in the brain and spinal cord. This disease is mainly characterized by crises of neurological dysfunction, after which a worsening of the neurological capacities of the patients occurs [3,4], affecting sexual function in 40–80% of women and 50–90% of men with MS [5,6,7,8].

In this sense, the processing of sexual stimuli can be altered in neurological disorders, which can prevent arousal, influence desire, reduce genital congestion, and even affect the patient’s sexual life and urinary continence [9]. Furthermore, the most common sexual dysfunctions (SD) in men with MS are erectile dysfunction, ejaculatory dysfunction and/or orgasmic dysfunction, reduced libido, and anorgasmia. On the other hand, the most frequently described SD in women is reduced libido and difficulty in reaching orgasm, reduced tactile sensations originating in the thigh and genital regions, and vaginal dryness with consequent dyspareunia [10,11].

A relationship has been described between psychological symptoms such as depression and anxiety and SD in patients with MS [12], which affects their quality of life [13]. Therefore, precise assessment instruments are needed that can help improve the therapeutic approach to this disease. For this, it is necessary to use patient-reported outcome measures (PROMs) that provide very valuable information, allowing the impact of subjective aspects of the pathology on the patient to be assessed, such as the impact on their quality of life, as this will influence clinical decision-making and the evaluation of the effectiveness of the treatment [14]. Thus, self-reported questionnaires play a key role.

In MS, it is more common to use specific questionnaires for the evaluation and classification of some of the most common signs and symptoms of people with MS. Some of these questionnaires include aspects of fatigue (Fatigue Impact Scale, FIS) [15], quality of life (Multiple Sclerosis Quality of Life 54, MSQoL-54) [16], and physical and psychological impact of the disease (Multiple Sclerosis Impact Scale, MSIS-29) [17].

The MS Intimacy and Sexuality Questionnaire is a questionnaire designed to assess SD symptoms in MS patients. Its first version was created in English by Dr. Foley and colleagues, who designed the tool to capture the multidimensional impact of MS on sexual function [18]. Since its initial development, the MSISQ-15 has been cross-culturally adapted and validated in seven additional languages: Polish, German, Italian, French, Turkish, Greek, and Spanish [19,20,21,22,23,24,25]. The MSISQ-15 divides SD into three dimensions: primary, resulting from neurological system function (questions 8, 12, 13, 14, 15); secondary, resulting from side effects of MS (questions 1, 2, 3, 4, 5); and tertiary, resulting from psychological, emotional, social, and cultural elements (questions 6, 7, 9, 10, 11). Patients rate each item with a Likert scale, which ranges from 1 (never) to 5 (always) [20].

## 2. Objectives

In order to improve future versions in other languages, it would be necessary to consider the structural characteristics and psychometric properties used in the versions already published. Therefore, the objective of this study was to analyze the structural characteristics and psychometric properties of the different language versions of the MSISQ-15, as well as the methodological quality, the quality of the evidence, and the criteria used, for good measurement properties.

## 3. Materials and Methods

### 3.1. Design and Protocol

A systematic review was conducted, encompassing articles published up to 27 October 2024, and registered in the PROSPERO database (PROSPERO ID: CRD42022344931), in accordance with the PRISMA statement [26] (see in Supplementary Materials) and the COSMIN guidelines [27].

### 3.2. Eligibility Criteria, Information Sources, and Search Strategy

The search was performed in the PubMed, SCOPUS, Web of Science, Dialnet, ScienceDirect, and CINAHL databases. The following MeSH terms were included with the Boolean operators AND/OR: “Multiple Sclerosis Intimacy and Sexuality Questionnaire” AND “sexual function” (“Sexual Dysfunction, Multiple Sclerosis” [Mesh]) OR “sexual function” [Title/Abstract]) AND “Surveys and Questionnaires” [Mesh] AND “Validation” [Title/Abstract] OR “Pelvic Floor Disorders” [Mesh]).

For this review, studies were included if they met the following criteria: they conducted cross-cultural validation of the MSISQ-15; involved adult participants (aged 18 or older) with multiple sclerosis who experience sexual dysfunction; and reported psychometric data for total and/or domain-specific scores of the MSISQ-15. Studies were excluded if the MSISQ-15 was only employed as an outcome measure, used to validate another instrument, or if the findings were inconclusive.

### 3.3. Study Selection

Records from various databases were imported into the Rayyan platform [28]. Initially, duplicate entries were removed, and then two independent reviewers (MMCP and ISH) screened the remaining records based on titles and abstracts. In cases of disagreement, a third reviewer (GMT) resolved the conflict. The studies that were ultimately selected were retrieved in full text for detailed content analysis and to determine their eligibility for inclusion in this review.

### 3.4. Data Extraction and Assessment of Risk of Bias

To collect information on the structural features and psychometric properties of each MSISQ-15 version, a detailed analysis of the different questionnaire adaptations was conducted. The methodological quality of each version was assessed using the risk-of-bias checklist from the COSMIN (Consensus-based Standards for the selection of health Measurement Instruments) guidelines, which are designed to support the selection of high-quality PROMs for both research and clinical practice [27,29].

For each version, the extracted structural characteristics included: title, year of publication, language/version, target population, sample size, age, sex, participant characteristics, setting, geographic location, pilot phase sample size, and number of participants per item. The psychometric properties evaluated were test–retest reliability, internal consistency, construct validity, and criterion validity. Each measurement property was then assessed individually according to the updated criteria for good measurement properties and rated as sufficient (+), insufficient (−), or indeterminate (?) [29,30].

### 3.5. Data Synthesis

Finally, the evidence was summarized, and the quality of the evidence was rated according to the Grades of Recommendation, Assessment, Development, and Evaluation (GRADE) approach [29].

## 4. Results

### 4.1. Study Selection Results

**Figure 1:**
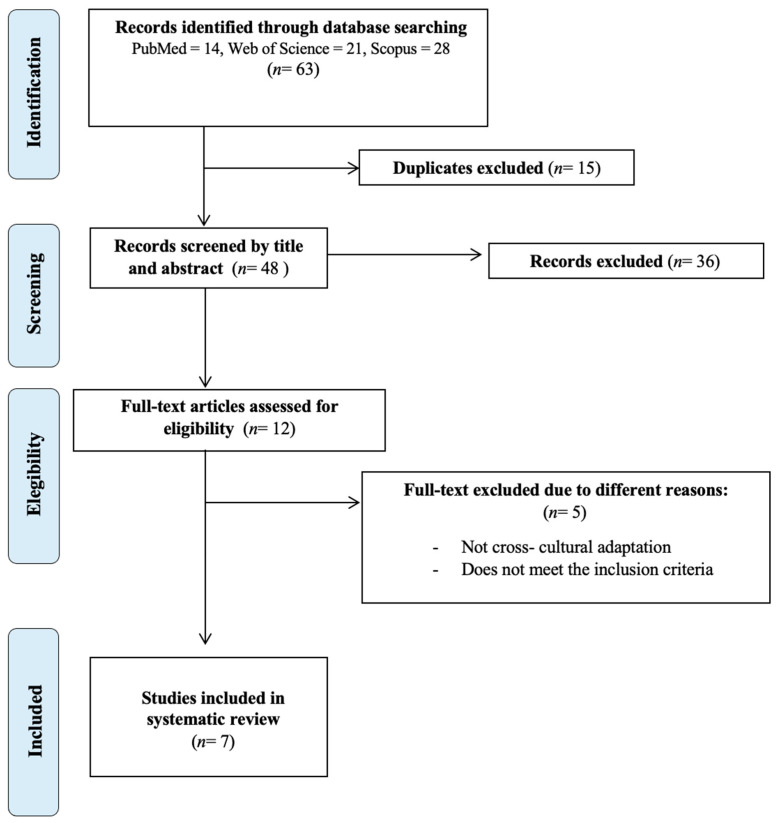
Flowchart for the selection of studies based on PRISMA.

Following the initial search in the PubMed, Scopus, and Web of Science databases, as shown in the flow chart of the selected studies (Figure 1), a total of 63 results were found. Excluding duplicates and after selecting articles by title and abstract, 48 were selected, of which 36 were excluded, and 12 full-text articles were selected for eligibility, of which five of them did not meet the inclusion criteria, did not present the results conclusively, did not include a validation phase, or were not a cross-cultural adaptation of the MSISQ-15.

Finally, a total of seven versions, adapted and validated to languages other than the original, were selected: Polish, Dutch, Italian, French, Turkish, Greek, and Spanish. Then, the structural characteristics of each one of them were analyzed (see Table 1).

### 4.2. Study Characteristics

Table 2 summarizes data corresponding to the psychometric properties of each questionnaire: test–retest, internal consistency, construct validity, and criterion validity.

### 4.3. Risk of Bias of Included Studies

#### 4.3.1. Structural Validity

None of the versions included an analysis of the structural validity of the MSISQ-15.

#### 4.3.2. Internal Consistency

Of the seven versions of the questionnaire, all included an internal consistency analysis using Cronbach’s alpha. All Cronbach’s alpha values were greater than 0.7, indicating a good internal consistency [29]. The highest value of Cronbach’s alpha was that of the Polish version, with a Cronbach’s α = 0.93 [23], while the lowest was the Italian version, α = 0.75 [21].

#### 4.3.3. Test–Retest Reliability

The intraclass correlation coefficient (ICC) was used in five of the six versions of the questionnaire, which calculates the test–retest reliability [19,20,21,22,23]. Only the Greek version [24] used Pearson’s correlation coefficient, while the Spanish version did not calculate the ICC [25]. All ICC values were greater than 0.7 and were, therefore, considered to have an acceptable reliability [31]. The highest ICC was found in the Turkish version, which was between 0.992 and 0.998 [19], and the lowest was the German version with a value of 0.88 [22]. The Italian version did not specifically define its ICC; it only specified that it was greater than 0.7 [21].

#### 4.3.4. Responsiveness

None of the versions included this measurement, which is the ability of a PROM to detect changes over time in the construct to be measured [27].

#### 4.3.5. Methodological Quality

Methodological quality was assessed according to the criteria of the COSMIN guidelines [27]. The quality of each study on a PROM must be assessed separately and can be classified as: very good, adequate, doubtful, or inadequate quality [27].

#### 4.3.6. Quality of Evidence

The GRADE system was used to assess the quality of the evidence [32], which considers the risk of bias, inconsistency, imprecision, and indirectness (see Table 3).

### 4.4. Synthesis of Results

**Figure 2:**
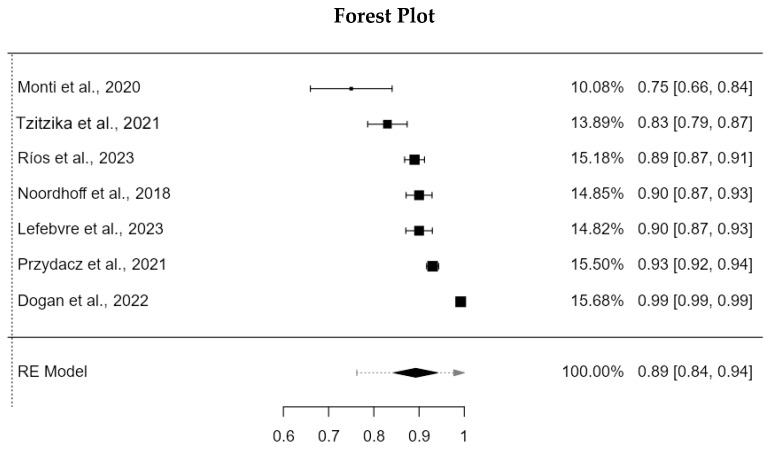
Forest plot with the Cronbach’s α coefficients of the MSISQ-15 [19,20,21,22,23,24,25].

The meta-analysis was performed with the 7 articles included in this review, as all the versions provided a Cronbach’s α value. The results indicate a cumulative Cronbach alpha coefficient of 0.893 (95%CI: 0.845–0.941; SE: 0.0245; Z: 36.4; *p* < 0.001) with a high heterogeneity (I2: 98.1%) (See Figure 2).

**Figure 3:**
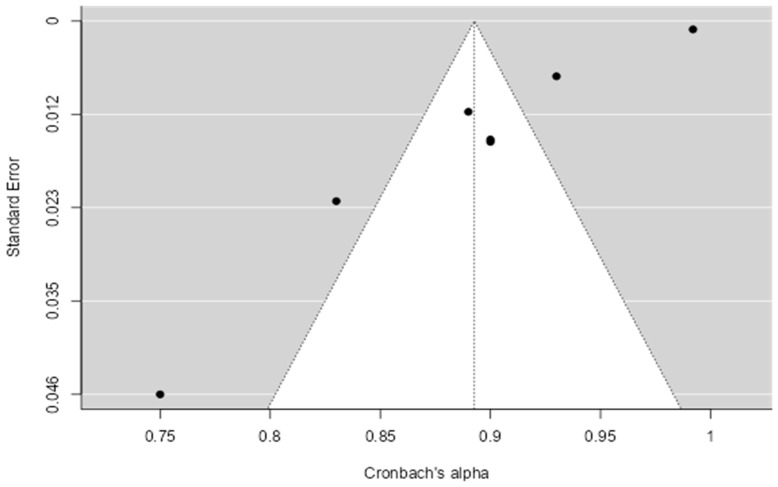
Funnel Plot with Cronbach’s α coefficients of the MSISQ-15.

The evaluation of publication bias through the Fail-safe N method yielded a value of 672.720 (*p* < 0.001), and Egger’s regression showed a value of −6.361 (*p* < 0.001). These results suggest a high consistency in the reliability of the measurements analyzed, despite the presence of significant heterogeneity between the studies (see Figure 3).

## 5. Discussion

This study aimed to carry out an analysis of the structural characteristics and psychometric properties of the MSISQ-15 questionnaire in all its versions, allowing them to be compared with each other and with the original version [18]. A total of seven versions of the MSISQ-15 were included: Polish [23], Dutch [22], Italian [21], French [20], Turkish [19], Greek [24], and Spanish [25].

In the validation phase, the version that included more patients was the Polish one with 227 patients [23]. On the contrary, the version that included fewer patients was the Italian one, with 67 [21]. The rest of the versions included: 208 patients in the Spanish version [25], 130 in the Turkish [19], 127 in the Greek [24], 102 in the Dutch [22], and 98 in the French [20] versions. The number of patients included in the cross-cultural adaptation was much lower than that of the original version, which included 6300 patients [18]. According to the COSMIN guidelines, a version of the MSISQ-15 questionnaire, which consists of 15 items, must include more than 150 participants to be rated as excellent. Only the Polish and Spanish versions [23,25] were rated as excellent, while the rest of the versions had more than 5 subjects per item, except for the Italian version [21], which included fewer than 5 subjects per item.

None of the versions considered structural validity, not even the original one. Internal consistency was included in all versions. However, none of them reached the values of the original version [18], which obtained a high internal consistency. Reliability was calculated in all versions using the ICC [19,20,21,22,23,24], except for the Spanish one, which was not calculated [25]. Nevertheless, in the remaining versions, it exceeded the value 0.7, or acceptable. Measurement error, which is important in decision-making and in the evaluation of change, was only calculated in the German, French, and Turkish versions [19,20,22]. Hypothesis testing was calculated in the German, Greek, Turkish, and Spanish versions [19,22,24,25], indicating a high risk of bias in the versions that did not present it [20,21,23]. Responsiveness was not calculated in any version due to the short follow-up that they adopted to ensure adequate test–retest analyses with no clinical interventions between the two measurements, and to minimize possible symptom changes.

### Strengths and Limitations of the Study

Regarding the results of the meta-analysis, the results of the first meta-analysis indicated that there was a high internal consistency in the questionnaire measures, with a mean Cronbach’s α of 0.89. However, a highly significant heterogeneity was observed, suggesting that there are differences in the measurements beyond random variability. Furthermore, evidence of publication bias was found using a funnel plot analysis, and confirmed using Egger’s test, which may be due to studies with negative and non-significant results, or those that do not support their preconceived theories, not being published or being incompletely reported.

## 6. Conclusions

In conclusion, the MSISQ-15 questionnaire has been adapted into 7 languages, each of them with good measurement properties, mostly rated as having a high and moderate methodological quality considering the COSMIN guidelines. The different versions of the MSISQ-15 are valid for use in the Polish, German, Italian, French, Turkish, Greek, and Spanish populations, and it will be necessary to adapt the questionnaire into other languages for use in patients with multiple sclerosis in other countries, considering the structural characteristics compiled in this review.

## Figures and Tables

**Table 1:** Structural characteristics of the questionnaires.

Questionnaire/Author, Year of Publication/ Version	Population/ Sample Size, Age, Group	Affected and Control Group	Setting/ Geographical Location	Target Population and Time Since Diagnosis	Number of Subjects–Phase Pilotage	Number of Subjects Per Item
The Multiple Sclerosis Intimacy and Sexuality Questionnaire (MSISQ-15): Validation of the Dutch version in patients with multiple sclerosis and spinal cord injury/Noordhoff et al., 2018/Dutch version [22]	*n* = 102 SCI: 53 MS: 49 Age: SCI: 41.3 ± 11.9 MS: 46.0 ± 10.1 Sex: SCI: Men: 12 ± 22.6 Women: 41 ± 77.4 MS: Men: 41 (83.7) Women: 8 (16.3)	AG: 102 CG: 50	NR	SCI and MS SCI: 10.1 ± 7.5 MS: 13.1 ± 11.7		total: 6.8 SCI: 3.53 MS: 3.29
The Multiple Sclerosis Intimacy and Sexuality Questionnaire (MSISQ-15): validation of the Italian version for individuals with spinal cord injury/Monti et al., 2020/ Italian version [21]	*n* = 65 Age: 40.4 ± 11.9 Sex: Men: 47 ± 72.3 Women: 27 ± 27.7		La Sapienza University of Rome and the Rehabilitation and Outcome Measures Assessment Association	SCI		4.33
The Multiple Sclerosis Intimacy and Sexuality Questionnaire (MSISQ-15): Validation and Cross-cultural Adaptation of the Greek Version in MS Patients/Tzitzika et al., 2021/Greek version [24]	*n* = 127 Age: MS: 45.57 ± 11.13 CG: 53.02 ± 9.36 Sex: MS: Men: 35 Women: 33 CG: Men: 34 Women: 25	AG: 68 CG: 59	Urology Department of the National Rehabilitation Centre of Athens	MS		8.46
The Multiple Sclerosis Intimacy and Sexuality Questionnaire (MSISQ-15): translation, adaptation, and validation of the Polish version for patients with multiple sclerosis and spinal cord injury/Przydacz et al., 2021/Polish version [23]	*n* = 227 SCI: 110 MS: 117 Age: SCI: 39.2 ± 9.9 MS: 44.2 ± 9.4 Sex: SCI: Men: 98 (89.1%) Women: 12 (10.9%) MS: Men: 35 (29.9%) Women: 82 (70.1%)	CG not included	Department of Urology of the Jagiellonian University Medical College, Krakow, Poland	SCI and MS SCI: 12.9 ± 10.9 MS: 9.2 ± 8.3	299 test phase	total: 15.33 SCI: 7.33 MS: 7.8
Validation of the French version of the Multiple Sclerosis Intimacy and Sexuality Questionnaire 15 Tools which help nurses assess the effect of perceived multiple sclerosis symptoms on sexual activity and satisfaction/Lefebvre et al., 2023/ French version [20]	*n* = 98 Age: 44.31 ± 11.33 Sex: Men: 30 Women: 68	AG: 51 CG: 51	Neurology department at the La Pitié Salpêtrière hospital in Paris, France	MS		6.53
The Multiple Sclerosis Intimacy and Sexuality Questionnaire (MSISQ): Validation of the Turkish version in patients with multiple sclerosis/Dogan et al., 2022/Turkish version [19]	*n* = 130 Age: 41.77 ± 10.84 Sex: Women 100%	AG:130	Neurology department of a university hospital	MS	30	8.66
Validation and cross-cultural adaptation of Multiple Sclerosis Intimacy and Sexuality Questionnaire-15 (MSISQ-15) into Spanish/Ríos et al., 2023/Spanish version [25]	*n* = 208 Age: 44.59 ± 9.788 Sex: Men: 73 Women: 135	CG not included	Multiple sclerosis associations in Spain	MS 11.685 ± 8.5052		13.86

SCI: Spinal Cord Injury, MS: Multiple Sclerosis, AG: Affected Group, CG: Control Group.

**Table 2:** Psychometric properties of the questionnaires.

Author/Version	Test–Retest Reliability	Internal Consistency	Construct Validity
Noordhoff et al., 2018/ Dutch version [22]	TS 0.88 (0.79–0.93) 1D 0.90 (0.84–0.94) 2D 0.75 (0.60–0.85) 3D 0.86 (0.77–0.92)	Cronbach’s α = 0.90	Indeterminate
Monti et al., 2020/ Italian version [21]	NE	Cronbach’s α = 0.75	MSISQ-15–SF-12 Mental health (r) Pearson: −0.360 MSISQ-15–SF-12 Physical health (r) Pearson: −0.219 MSISQ-15–SCIM SR Self-care (r) Pearson: −0.033 MSISQ-15–SCIM SR Management of respiration and sphincter (r) Pearson: −0.036 MSISQ-15–SCIM SR Mobility (r) Pearson: −0.003 MSISQ-15–Total SCIM SR: −0.028
Tzitzika et al., 2021/ Greek version [24]	TS 0.83 1D 0.84 2D 0.77 3D 0.86	Cronbach’s α = 0.83	MSISQ-15–IIEF (r): −0.57 MSISQ-15–FSFI (r): −0.60
Przydacz et al., 2021/ Polish version [23]	TS 0.91 (0.80–0.95) 1D 0.93 (0.82–0.97) 2D 0.78 (0.70–0.86) 3D 0.87 (0.79–0.93)	Cronbach’s α = 0.93	MSISQ-15–IIEF-15 (r) Pearson Test phase: −0.487 MSISQ-15–IIEF-15 (r) Pearson Retest phase: −0.456 MSISQ-15–PISQ-31 (r) Pearson Test phase: −0.709 MSISQ-15–PISQ-31 (r) Pearson Retest phase: −0.688
Lefebvre et al., 2023/ French version [20]	TS 0.90 (0.63; 0.98) 1D 0.91 (0.65; 0.98) 2D 0.30 (−0.24; 0.76) 3D 0.93 (0.74; 0.98)	Cronbach’s α = 0.90	Indeterminate
Dogan et al., 2022/ Turkish version [19]	TS 0.998 1D 0.992 2D 0.990 3D 0.994	Cronbach’s α = 0.992	MSISQ-15–MSQOL-54 (r) Pearson correlation 1D: −0.647; 2D: −0.706; 3D: −0.703; Total: −0.763 MSISQ-15–FSFI (r) Pearson correlation 1D: −0.776; 2D: −0.594; 3D: −0.655; Total: −0.754 MSISQ-15–PSIQ-12 (r) Pearson correlation 1D: −0.741; 2D: −0.678; 3D: −0.782; Total: −0.798
Ríos et al., 2023/Spanish version [25]	NE	Cronbach’s α = 0.89	MSISQ-15–FSH (rho) Spearman 1D: (−0.53); 2D: (−0.31); 3D: (−0.42); Total (−0.52) MSISQ-15–FSM-2 (rho) Spearman 1D: (−0.65); 2D: (−0.27); 3D: (−0.32); Total (−0.55) MSISQ-15–EAD-13 (rho) Spearman 1D: (−0.14); 2D: (−0.10); 3D: (−0.08); Total (−0.14) MSISQ-15–MusiQol (rho) Spearman 1D: (−0.25); 2D: (−0.35); 3D: (−0.38); Total (−0.039)

TS: total score, 1D: primary domain, 2D: secondary domain, 3D: tertiary domain, NE: not evaluable.

**Table 3:** Analysis of the rating of the psychometric properties, methodological quality, and quality of evidence.

PROM	Version	Structural Validity (Rating)	Internal Consistency (Rating)	Reliability (Rating)	Measurement Error (Rating)	Hypotheses Testing (Rating)	Responsiveness (Rating)
Przydacz. et al., 2021 [23]	Polish	NR	Sufficient	Sufficient	NR	NR	NR
Methodological quality Risk of bias		NR	Very good	Adequate	NR	NR	NR
Quality of evidence		NR	High	Moderate	NR	NR	NR
Noordhoff et al., 2018 [22]	Dutch	NR	Sufficient	Sufficient	NE	Sufficient	NR
Methodological quality Risk of bias		NR	Very good	Doubtful	Very good	Very good	NR
Quality of evidence		NR	Moderate	High	Moderate	Moderate	NR
Monti et al., 2020 [21]	Italian	NR	Sufficient	Sufficient	NR	NR	NR
Methodological quality Risk of bias		NR	Very good	Doubtful	NR	NR	NR
Quality of evidence		NR	High	Moderate	NR	NR	NR
Lefebvre et al., 2023 [20]	French	NR	Sufficient	Sufficient	Sufficient	NR	NR
Methodological quality Risk of bias		NR	Very good	Adequate	Adequate	NR	NR
Quality of evidence		NR	Moderate	Moderate	High	NR	NR
Dogan et al., 2022 [19]	Turkish	NR	Sufficient	Sufficient	NE	NE	NR
Methodological quality Risk of bias		NR	Very good	Doubtful	Adequate	Very good	NR
Quality of evidence		NR	High	High	Moderate	Moderate	NR
Tzitzika et al., 2021 [24]	Greek	NR	Sufficient	Sufficient	NR	Sufficient	NE
Methodological quality Risk of bias		NR	Very good	Adequate	NR	Very good	Inadequate
Quality of evidence		NR	High	Moderate	NR	Moderate	Moderate
Ríos et al., 2023 [25]	Spanish	NR	Adequate	NR	NR	NR	NR
Methodological quality Risk of bias		NR	Very good	NR	NR	NR	NR
Quality of evidence		NR	Moderate	NR	NR	NR	NR

NR: not reported; NE: not evaluable.

## Data Availability

The original contributions presented in this study are included in the article/Supplementary Materials. Further inquiries can be directed to the corresponding author.

## Supplementary Materials

The following supporting information can be downloaded at: https://www.mdpi.com/article/10.3390/diagnostics15222836/s1, PRISMA 2020 Checklist. Reference [33] was cited in Supplementary Materials.
